# Damage evolution and fracture behavior of different materials specimens containing a central hole subjected to local loading

**DOI:** 10.1038/s41598-022-21020-x

**Published:** 2022-10-03

**Authors:** Dongliang Ji, Hongbao Zhao, Hui Cheng, Xiuhan Yang, Lina Ge

**Affiliations:** 1 School of Energy and Mining Engineering, China University of Mining and Technology (Beijing), Beijing, 100083 China; 2 Department of Civil Engineering, University of Ottawa, Ottawa, K1N 6N5 Canada; 3 School of Civil Engineering, Liaoning Technical University, Fuxin, 123000 China

**Keywords:** Natural hazards, Solid Earth sciences

## Abstract

The strength of the different materials specimens containing a central hole subjected to varying loading areas constitutes lots of underground engineering such as entry arrangement and mining process. In this study, the failure resulted from micro-fracturing in the specimen, which can be characterized by the crack propagation path if the damage events are monitored by using Digital Image Correlation (DIC), infrared thermal imager and high-speed camera. The experimental results demonstrate that there are three different types of typical failure modes for specimens with central holes according to the loading areas. The evolution of the temperature field is shown for various loading areas, the smaller loading area, the greater the stress concentration, and the more pronounced the thermodynamic features. The temperature field can also be associated with material properties in addition to loading area. Additionally, failure around the hole with redistributed stress has been observed, and strain energy density (SED) can help explain the failure mechanisms. The progressive damage process, which takes into account the heterogeneity in elastic modulus and rock strength characteristic, is demonstrated by developing a constitutive model that uses the uniaxial compression and Brazilian disc tests to parameterize it. By comparison with plastic zone, the proposed constitutive model is used to quantitatively evaluate the accumulation of damage. Failure mechanisms are established based on this work and are anticipated to be extensively utilized in engineering applications.

## Introduction

In the construction of rock-mass engineering and backfill mining, coal rock masses and concrete are categorized as the common discontinuous and heterogeneous engineering medias, which contain numerous pores and fissures, and many tests have verified that damage initiate and propagate from weak parts, resulting in catastrophic failure. During the advancing of work face, the lower roadway or larger holes are subjected to varying upper loading. Therefore, to consider the initiation location of crack and progressive failure process is essential in many foundation engineering.

In previous studies, fracture process of rock specimens containing the hole was mainly concerned, the gradual evolution of the strain localization region revealed the crack propagation process and the fracture mode was mainly associated with the inclination angle of fissures^1,2^. Tensile cracks were dominant when the inclination angle was small, but with the increase of the angle, the shear fractures mostly occurred in the specimen^3,4^, while it also was found that shear cracks were dominant, followed by tensile cracks, and cracks were smallest in the mixed mode^5,6^. Moreover, the evolution process of shearing fracture can also be demonstrated through real-time deformation field^7^, the growth of original cracks and new cracks were closely related to acoustic emission (AE) events from the perspective of acoustic emission^8,9^. In addition, the progressive damage and failure process of the shear mode can be analyzed experimentally and numerically^10–12^.

There are several studies on influence of preexisting holes on the properties of the rock specimen including stimulative and inhibitory effects. The effective elastic modulus firstly decreased and then gradually increased with the size of hole in the specimen increasing, and a statistical damage constitutive model can be established^13^. The failure mode, location and strength of pores of different shapes were mainly affected by lateral pressure^14^, with the increase of lateral pressure coefficient, the failure was further intensified, which mostly occurred in the roof and floor of the central pore. The most important conclusion was that the deformation and failure process of the rock-mass with holes can be divided into two stages: rock bridge coalescence and overall instability^15–17^. When the number of holes switched from 1 to 2, the failure processes of rock disc with hole and that with eccentric hole were firstly compared and analyzed^18,19^, meanwhile, the accumulate strain were more likely to appear^20^. By contrast, the original cracks exhibited inhibition effects to certain extent on the new hole in terms of propagation^21^. An increase in the number of holes in the specimen from two to more, it was found that the occurrence of macro fracture was still due to the coalescence of cracks generated at the edge of the pores^22^. By comparing finite element model results with experimental results, both of which in reasonably good agreement with each other in terms of initial cracking, expansion direction and expansion pressure^23,24^. When it came to rock structure crevice group, the mechanical behaviors of sandstone specimens with different fissure angles, ligament length and fissure length under uniaxial compression were analyzed^25^. Under coupling static and dynamic loading, it was possible under suitable conditions to produce initial fracture^26^, considering the stress concentration before and after pre-shearing, the initially produced static load and the far field strain could promote the impact damage of rock^27^. In addition, influence of eccentric load^28^, different stress gradients^29^ on mechanical properties and damage process was conducted, indicating that local loading decreased the value of peak stress, crack initiation stress. The finding in Zhang et al.^30^ suggested localization effect of local frequent dynamic disturbance on micro-structure evolution of coal-rock. Such studies simplified the local loading and hence failed to reflect the realistic condition of the stress state. Thus, the effect of local loading on specimens containing the central hole remains to be better clarified.

The experimental approaches based on DIC have been used by many researchers. DIC was employed to provide displacement and stress conditions in full-field^31^. An improved shape and methodological approach was put forward with which was employed to evaluate and improve the accuracy of DIC strain measurements^32^. The quality of strain field obtained with different processing parameters is also assessed^33^. Infrared radiation, a characterization of temperature field, was more and more widely used in the failure progress of rock. As temperature change occurred at the moment of rock failure, thermal infrared radiation characteristics were employed to display the influence of water-bearing characteristics on the fracture process^34^, the response to dynamics^35^ and the evolution of surface deformation field^36^. Additionally, it was verified that the temperature field and stress field were consistent in the dynamic evolution process under excavation of the circular tunnel^37^.

Moreover, previous studies have mainly focused on coalescence of cracks, the dynamic mechanical model under static and dynamic loading, and mechanical instability and failure of rock mass. However, enough attention had not been paid to failure problem under local loading due to the hole and the hole distribution characteristic. Therefore, it is of great significance to analysis the progressive failure process under local loading, to clarify the failure mechanism. In this paper, first, specimens containing the central hole were fabricated to investigate failure process, a series of conventional experiments were conducted under varying loading areas, and introduced characterization parameters for DIC and temperature field. Then, the relationships of the evolution of deformation field, temperature field with respect to mechanical characteristics were discussed, respectively. Furthermore, a self-defined constitutive model was employed to further the understanding on the effect of local loading on fracture mechanism of progressive failure process.

## Materials and methods

### Materials and preparation

**Figure 1:**
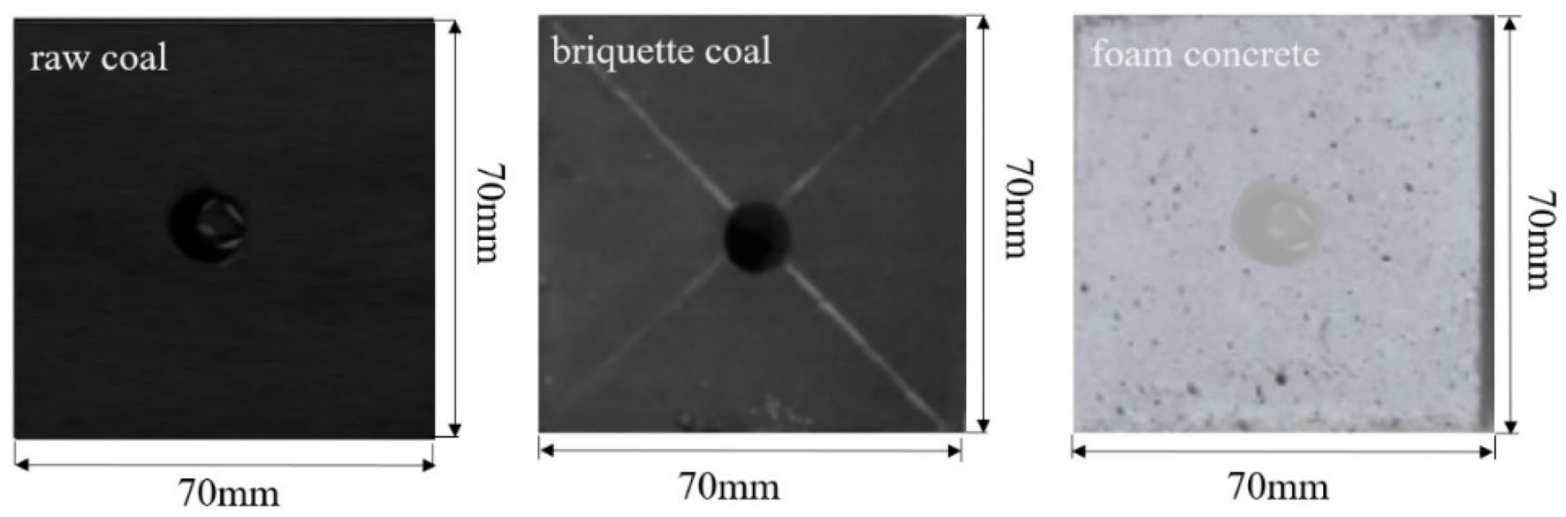
Schematic of specimens.

For the laboratory study, three kinds of materials were chosen as the test objects, which were processed accordance with the recommended test methods and requirements of International Society for Rock Mechanics. The raw coal was taken from Hequ coal mine and prepared with the damp processing, the well-proportioned briquette coal material with content of 10:2.4:1.6 of coal, cement and water was loaded into the mold, and then the mold was retreated on the press with a pressure of 20 MPa for 30 min. The briquette specimen provided the match with strength obtained by the test. The foam concrete was most commonly used in mine filling, which was made by pressing cement mixed with aggregate, admixture and water in appropriate proportions, maintained in wet conditions. Then, high-pressure water jet cutting technology was used to produce hole in three specimens, the specimens prepared were shown in Fig. 1.

**Figure 2:**
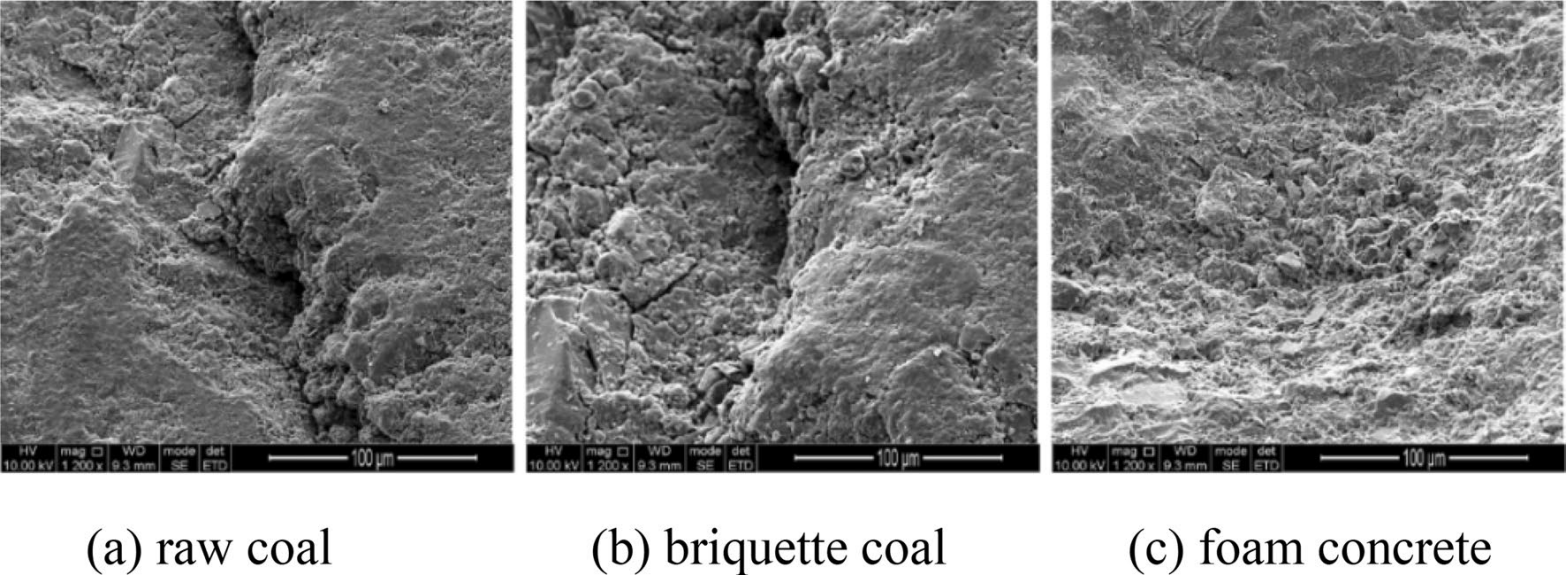
Microscopic structure of specimens.

Three kinds of specimens were all made into 70 mm × 70 mm × 70 mm cube, and a vertical through hole with diameter of 8 mm was drilled at the center of each specimen. The surface roughness as well as end surface perpendicularity of the specimens were less than 0.02 mm and 0.01 mm, respectively, the basic parameters of specimens were shown in Table 1. In order to understand the formation of microdefects, growth of the holes and the distribution of connected holes in the internal structure of the specimen, the small specimens were fixed on a conductive adhesive and sprayed gold at 10 mA for 30 s. Then surface morphologies were characterized using a JSM-7800 F field emission scanning electron microscope (SEM) at an accelerating voltage of 3 kV, as shown in Fig. 2. Raw coal is more compact, and form concrete contains spaces between grains.

**Table 1:** Main parameters of specimens.

Specimens	Raw coal	Briquette coal	Foam concrete
Length of the side (mm)	70	70	70
The internal diameter (mm)	8	8	8
Proportion	On-site sampling wet processing	Coal: cement: water 10:2.4:1.6	Cement: aggregate: water: foaming agent 540:700:620:1
Pore radius distribution (μm)	0.001–10	0.01–100	0.07–1000
Number	20	20	20

### Experimental apparatus and procedure

**Figure 3:**
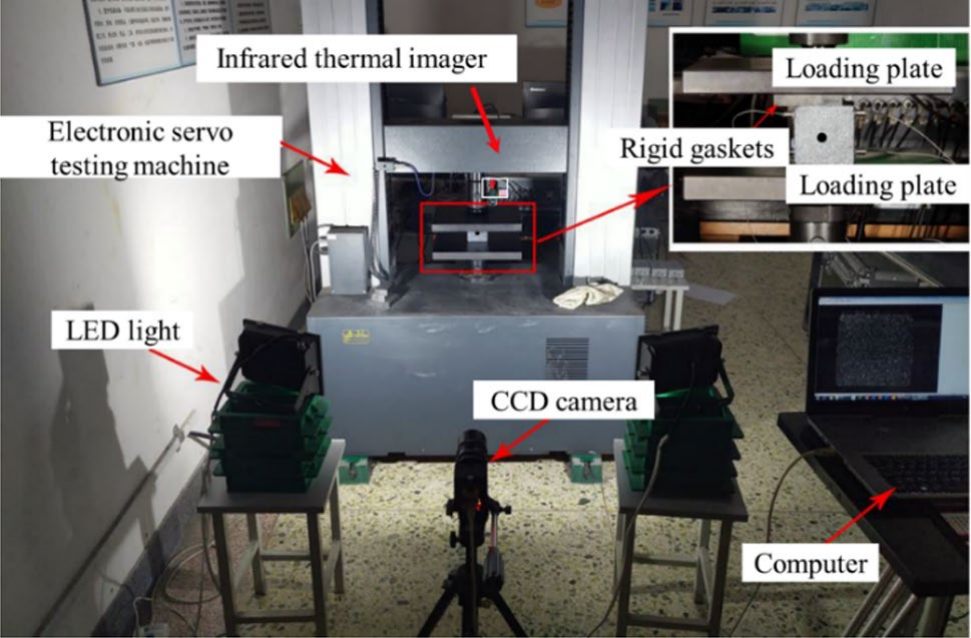
Actual picture of testing system.

Electronic servo testing machine (WDW-300E) was used to compress the specimen with varying loading areas, the measurement accuracy was less than ± 0.5% and its resolution was 0.001 mm, the axial force was applied on the specimen in pressure-controlled conditions with a loading rate of 0.5 MPa/s until failure occurred. Meanwhile, DIC was employed to directly observe the displacement and deformation of the specimens. Microstructure was captured by a high-speed camera. And in order to display the temperature field evolution in real time, the instantaneous surface temperature data of the three kinds of specimens under peak stress were collected by infrared thermal imager, and a total of 136 pixels points on the surface of each specimen were monitored. With the help of the above devices, it was helpful to reflect the mechanical characteristics and triggered failure process of specimen containing the central hole by the specific quantitative indexes. The actual picture of testing system was shown in Fig. 3.

**Figure 4:**
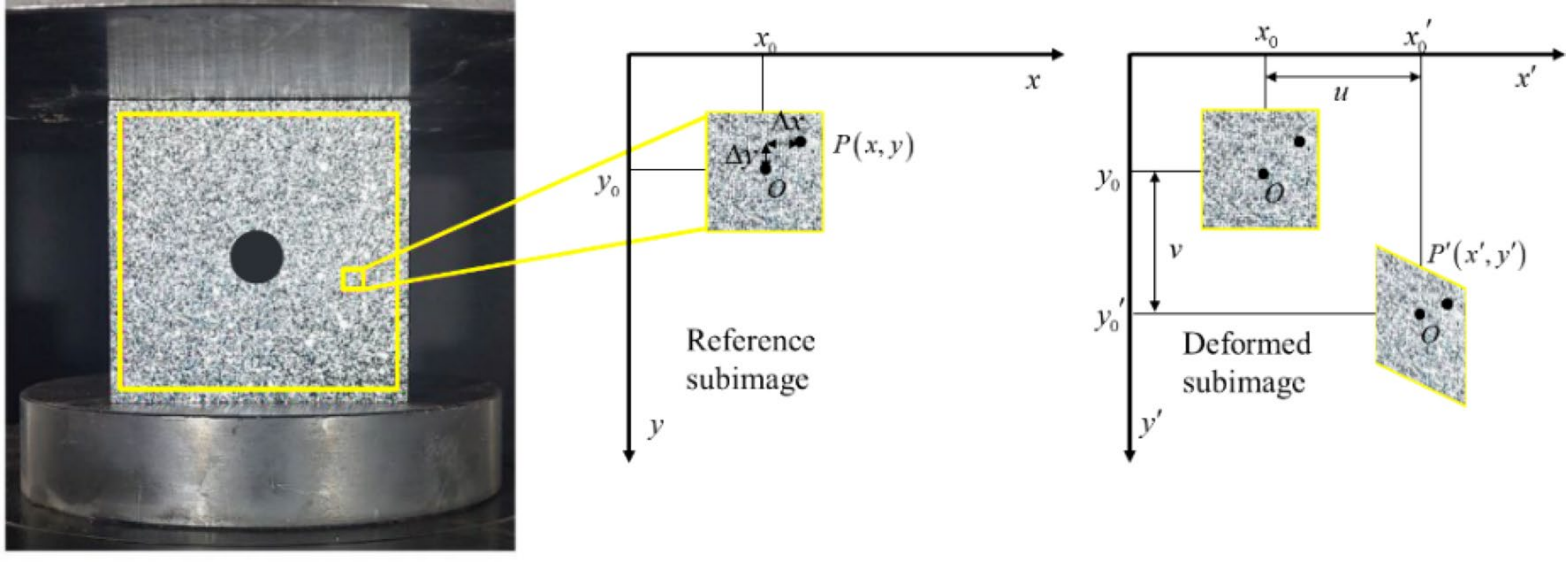
Schematics of the reference and deformed subimages.

Based on DIC measurements principle, DIC was used to identify the same pixel points established to provide deformed images such as zero-normalized cross-correlation^38^. DIC method was divided into the following steps: (1) a square subset (2*N* + 1) × (2*N* + 1) in the deformed image is detected under the condition of maximum the correlation coefficient; (2) the displacement components corresponding to the position of the target subset can be determined; (3) The point $P^{\prime}(x^{\prime},y^{\prime})$ after deformation with respect to the coordinate (*x*_0_, *y*_0_) was shown as in Fig. 4:

1 $$ \left\{ {\begin{array}{*{20}c} {x^{\prime} = x_{0} { + }x + r + \frac{\partial r}{{\partial x}}\Delta x + \frac{\partial r}{{\partial y}}\Delta y} \\ {y^{\prime} = y_{0} + y + p + \frac{\partial p}{{\partial x}}\Delta x + \frac{\partial p}{{\partial y}}\Delta y} \\ \end{array} } \right. $$

where *r, p* are the displacement components in *x, y* direction, respectively. $\Delta x$ and $\Delta y$ are the distance from point P to point O, $\frac{\partial r}{{\partial x}}$, $\frac{\partial r}{{\partial y}}$, $\frac{\partial p}{{\partial x}}$ and $\frac{\partial p}{{\partial y}}$ are the gradients of displacement components.

### Loading methods

**Figure 5:**
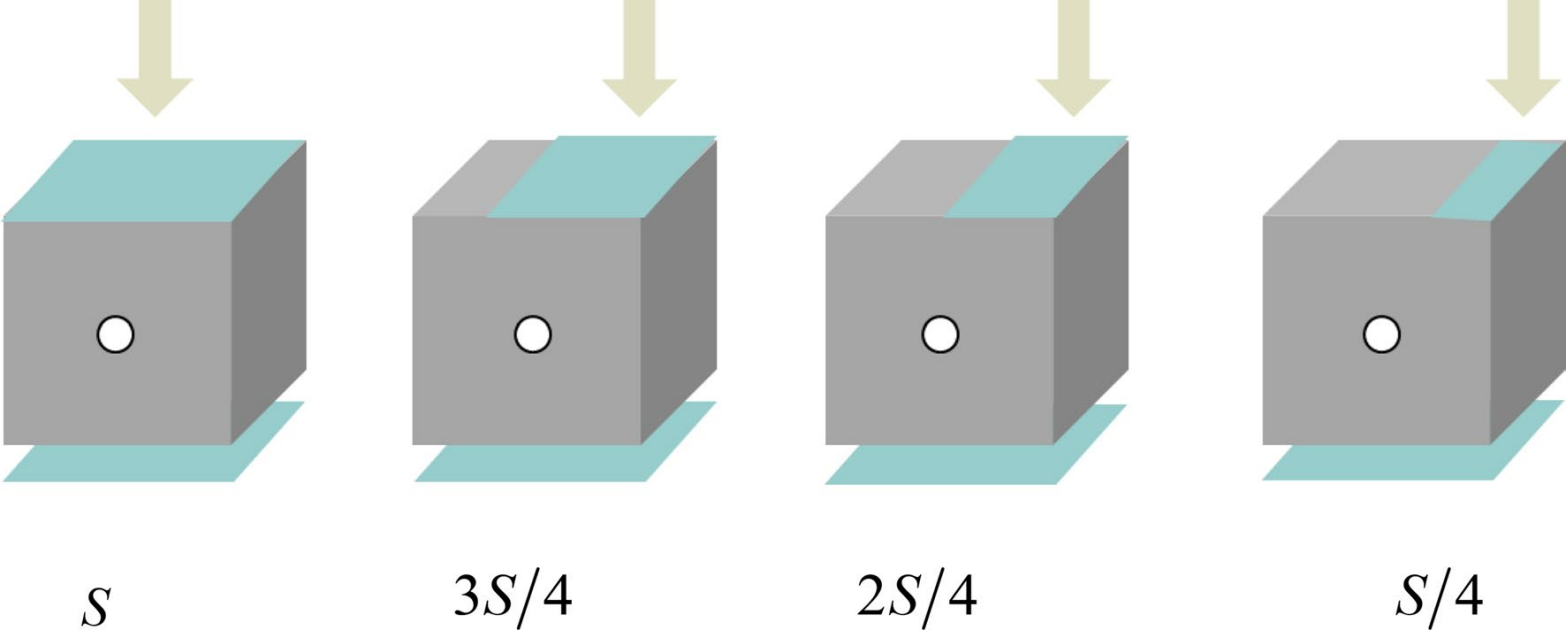
Schematic of loading modes.

In order to rule out the differences caused by its own unique and complicated pore space structure, each experiment was repeated for 5 times in the test, and the loading test device was used to carry out loading tests on three kinds of specimens. To assess the influence of loading area on the existing hole, the loading areas were $S$, ${{3S} \mathord{\left/ {\vphantom {{3S} 4}} \right. \kern-\nulldelimiterspace} 4}$, ${{2S} \mathord{\left/ {\vphantom {{2S} 4}} \right. \kern-\nulldelimiterspace} 4}$ and ${S \mathord{\left/ {\vphantom {S 4}} \right. \kern-\nulldelimiterspace} 4}$, respectively, as shown in Fig. 5. ($S$ represents the total area of one surface of the sample).

## Result and discussions

### Crack propagation

**Figure 6:**
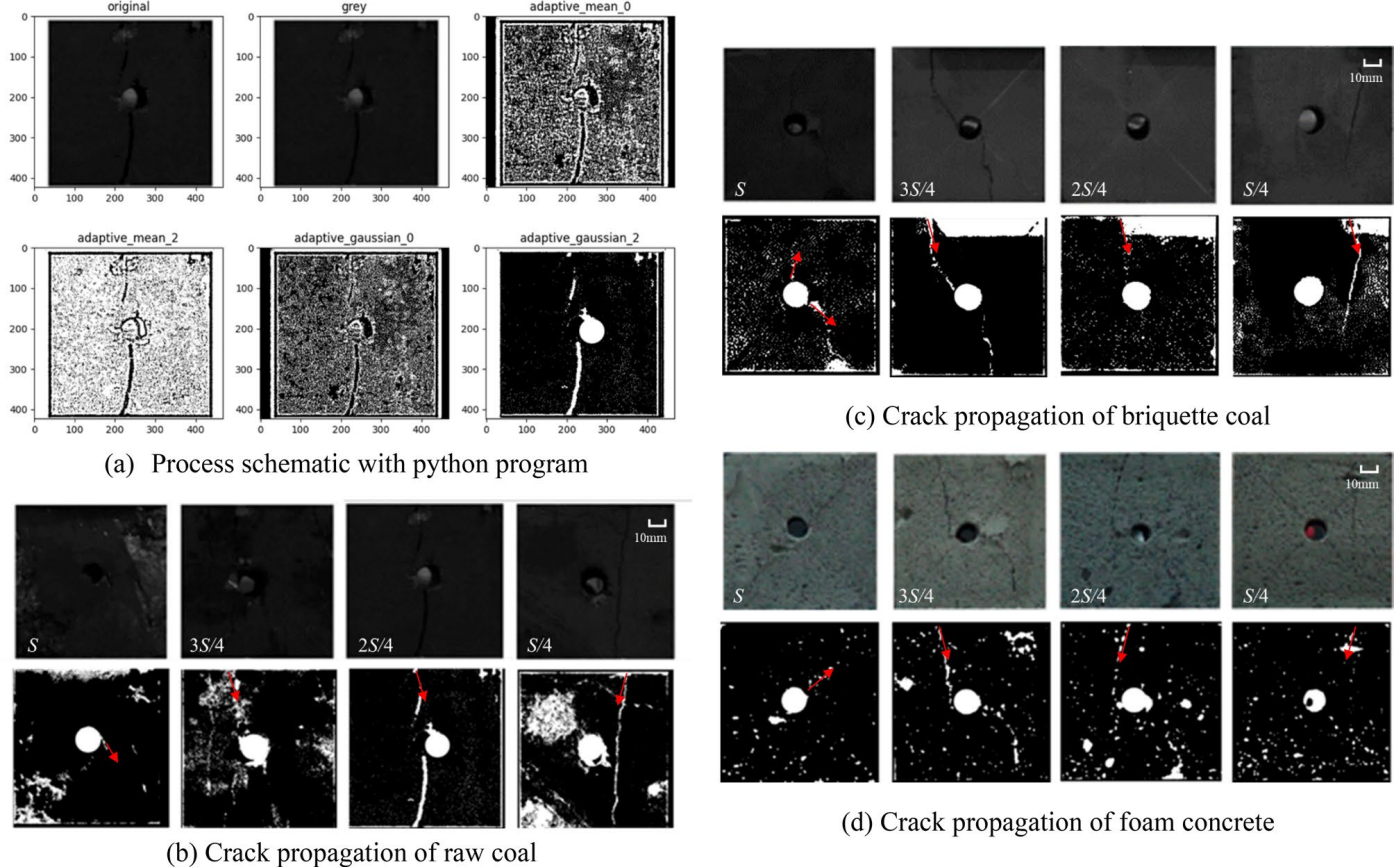
Crack propagation models with different materials (Directions of crack propagation are indicated in red arrows).

The tests were instrumented with a Fastcam SA-Z high-speed digital camera in order to capture the progressive failure process of the specimens, and obtained image were processed by python-compiled programs including gray, adaptive mean and adaptive Gaussian, and then cracks were identified, as shown in Fig. 6.

There were three modes of failure differentiated by loading methods. When the loading area was at $S$, stress concentration occurred around the central hole of the specimen, and cracks were generated from the hole of specimen, then expanded towards the boundary direction, finally forming macroscopic failure. Under the loading areas were at ${{3S} \mathord{\left/ {\vphantom {{3S} 4}} \right. \kern-\nulldelimiterspace} 4}$, ${{2S} \mathord{\left/ {\vphantom {{2S} 4}} \right. \kern-\nulldelimiterspace} 4}$, cracks started from the loading critical location and developed in the vertical direction, then intersected with the central hole, meanwhile, crack propagated from the central hole of specimen and eventually elongated into an arc shape. Under the loading area was at ${1 \mathord{\left/ {\vphantom {1 4}} \right. \kern-\nulldelimiterspace} 4}S$, cracks first appeared at the critical location of local loading and extended downward directly, the cracks grew discontinuously, no cracks were generated at the central hole.

Overall, similar failure characteristics were observed in all three specimens under different loading areas. Therefore, it can be obtained that loading area had a significant influence on the crack growth mode. Under the full-area loading, cracks first appeared around the hole, then grew, and with the decrease of the local loading area (${{3S} \mathord{\left/ {\vphantom {{3S} 4}} \right. \kern-\nulldelimiterspace} 4}$, ${{2S} \mathord{\left/ {\vphantom {{2S} 4}} \right. \kern-\nulldelimiterspace} 4}$), cracks first appeared at the critical loading location, and then gradually connect with the cracks produced at the central hole, when the loading area was small, cracks also first appeared at the critical loading location, but no cracks passed through the macroscopic central hole.

### Characteristics of deformation field

**Figure 7:**
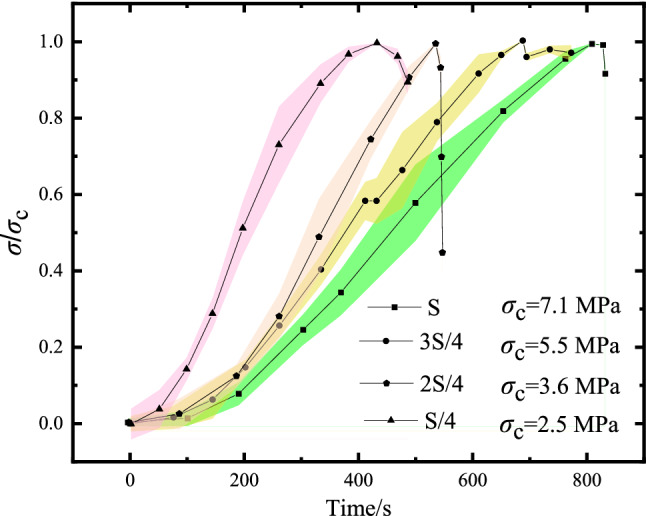
Stress-time curves under different loading areas.

Figure 7 summarized the mechanical properties of foam concrete under different loading areas in our experiments. It can be found that the loading area significantly affected the shape of stress-time curves. Under the lowest loading area, evident shortened time reaching to peak stress ($\sigma_{c}$ is the peak stress corresponding to different loading areas) can be found in the stress-time curve (e.g., ${S \mathord{\left/ {\vphantom {S 4}} \right. \kern-\nulldelimiterspace} 4}$). With the increase of loading area, the time reaching to peak stress of curves became greater.

The localized field under different loading area undergo an obvious deformation prior to failure. The evolution of deformation field was analyzed with increasing of loading stress, meanwhile strain field was obtained by MATLAB software. Statistical indicator can be conveniently accommodated into spatial distribution of deformation field values^39^. The deformation concentration occurred due to uneven deformation of sample, at which the slope of $S_{{\text{w}}}$^40,41^ appear a turning point, implying the initial localization of rock deformation. $S_{{\text{w}}}$ was defined as

2 $$ \left\{ {\begin{array}{*{20}l} {S_{{\text{w}}} = W_{{\text{s}}} * S^{\prime}} \hfill \\ {S^{\prime} = S^{\prime}(X_{k} ) = \sqrt {\frac{1}{n - 1}\sum\limits_{k = 1}^{n} {(X_{k} - \overline{X})^{2} } } } \hfill \\ {W_{{\text{s}}} = S^{\prime}(X_{k}^{ * } ) = \sqrt {\frac{1}{n - 1}\sum\limits_{k = 1}^{n} {(X_{k}^{ * } - \overline{X}^{ * } )^{2} } } } \hfill \\ \end{array} } \right. $$

$$ \overline{X}{ = }\frac{1}{n}\sum\limits_{k = 1}^{n} {X_{k} } \quad X_{k}^{ * } = X*B $$

where $W_{{\text{s}}}$ is the weighting coefficient of deformation localization, $S^{\prime}$ is the variance of deformation field, $X_{k}$ is the strain value of each point, $\overline{X}$ is the average value of $X_{k}$, $X^{ * }$ is the convolution of a matrix of strain values and a matrix B.

**Figure 8:**
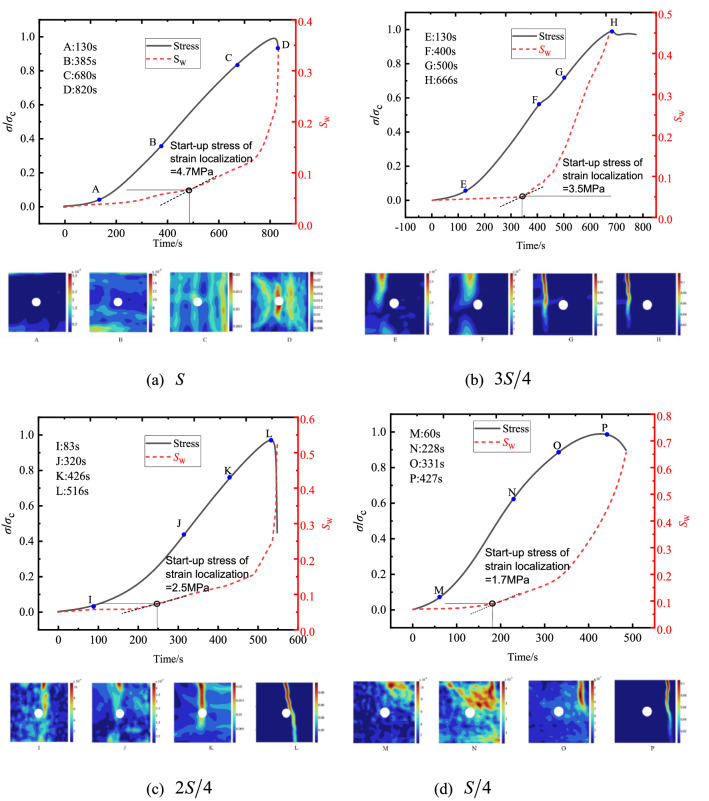
Strain fields at different loading stages.

The deformation Field on surface of foam concrete at different times were compared and analyzed. As shown in Fig. 8a, stress was low and closed to zero prior to Point A, increase in loading stress cannot result in a remarkable change of deformation field. After Point A, stress increased significantly, as a result, the stress concentration appeared in the sample. It was obvious that slope change of $S_{{\text{w}}}$ took place at Point C, this meant Point C can approximate the point where start-up stress began. After Point C, value of $S_{{\text{w}}}$ increased significantly with increasing of the stress. Given the sharp gradient of $S_{{\text{w}}}$, it can be concluded that macroscopic cracks have formed there, which was in agreement with the final failure observed in experiment, then the presence of cracks can be identified from the deformation field. Comparison with case of $S$, as shown in Fig. 8b and c, with the increase in loading stress, fracturing developed downwards and connected to the central hole. Afterwards, the fracture eventually went through the sample due to continuous development of the cracking, and start-up stresses of strain localization were 3.5 MPa and 2.5 MPa, respectively. For the case of ${S \mathord{\left/ {\vphantom {S 4}} \right. \kern-\nulldelimiterspace} 4}$ (Fig. 8d), cracks first appeared at the critical loading location, and the exist hole do not generate any failures, start-up stress of strain localization was 1.7 MPa. It was denoted that the exact position and propagating direction of the crack can be determined by using DIC.

### Evolution of surface deformation energy density

Deformation energy density was also an index used to analyze the effects of stability on potential failure^42^.

3 $$ u = \frac{E}{2}\left( {\varepsilon_{1}^{2} + \varepsilon_{2}^{2} - 2v\varepsilon_{1} \varepsilon_{2} } \right) $$

where $E$ is elasticity modulus, $\varepsilon_{1}$, $\varepsilon_{2}$ are first principal strain and second principal strain, respectively, $v$ is Possion's ratio, $u$ is deformation energy density.

**Figure 9:**
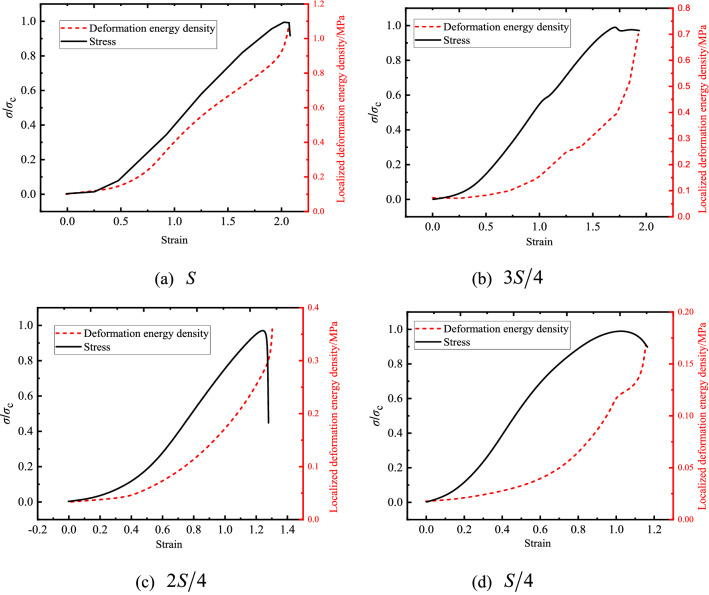
Localized deformation energy under different loading areas.

Considering that rock failure was induced by varying loading areas, deformation energy density was investigated on surface. Figure 9 presented the deformation energy corresponding to increasing loading stress with decreasing values of loading areas. It can also be observed that the deformation energy density was approximately zero at initial stage, then it increased significantly especially in the later period, which was consistent with DIC results. The results indicated that the sample failure occurred, and a part of deformation energy density was released in the failure process. It was important to note that values of deformation energy density decreased with decreasing of loading areas, which was attributed to energy inputted under different loading areas, the energy distribution represented the superposition of the static stress field and hole effect, it can be inferred that the less loading area is, the less deformation energy density is.

### Analysis of temperature field

**Figure 10:**
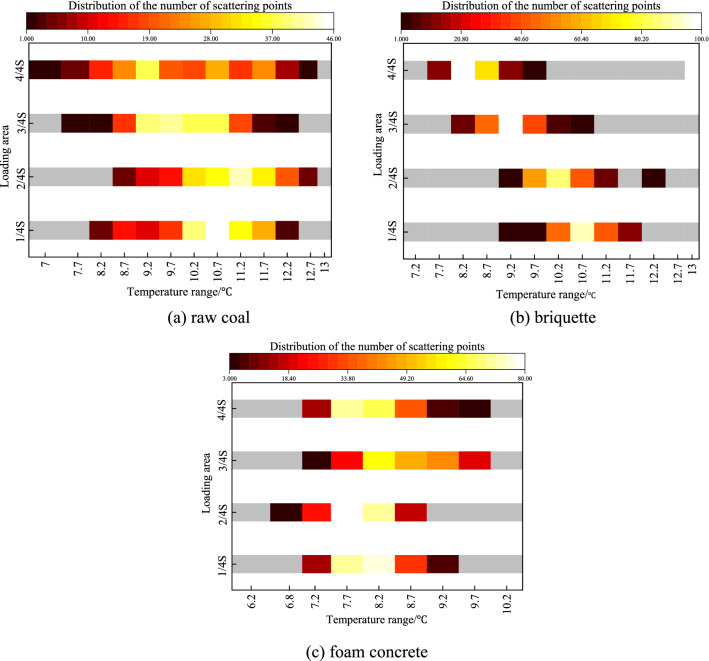
Number of pixels points in different temperatures.

The evolution characteristics of temperature field on the specimen surface corresponded well with the loading methods, and forming different thermodynamic environments. The failure of specimens can be deemed to be caused by the growth and coalescence of internal cracks during the loading process^43^. In this test, infrared thermal imager together with its supporting software was used to monitor the temperature changes of the specimen surfaces, it provided important infrared precursor of rock fracture and destabilization. The number of their distribution in different temperature ranges was shown in Fig. 10.

Images in Fig. 10 showed the surface temperature fluctuated and varied inconsistently in different areas after loading progress. The results indicated that increase of number value of peak temperature became more evident with decreasing of loading area for three kinds of specimens, which was because local loading cause stress concentration in a short time, and the higher the degree of stress concentration, the more obvious the regional thermodynamic phenomenon would be, which was agreement with results in literature^44^. Therefore, the stress concentration under local loading can be directly reflected through temperature distribution range and temperature field change. In addition, it can be seen that under the same loading conditions, the surface temperature of raw coal specimens was significantly higher than that of briquette and foam concrete specimens, which was due to the large strength and densest structure of raw coal.

Entropy was usually used to describe the uniformity of distribution of any kind of energy^45^. The thermal image corresponding to a certain loading time of the specimen was ranked according to the maximum difference in temperature. Different loading conditions may cause changes in the surface temperature field of the specimen, which was mainly reflected in the change of entropy value. It may lead to an increase in temperature within the deformation concentration zone, the temperature distribution range of the infrared thermal radiation field became wider, and the difference in surface energy of the specimen became larger^46,47^.

Entropy is normalized and represented by $H$ ($H = 0\sim 1$), as shown in the following formula:

4 $$ \left\{ {\begin{array}{*{20}c} {I = - \sum\limits_{n = 1}^{N} {P_{{\text{n}}} \lg P_{{\text{n}}} } } \\ {H = I/\lg N} \\ \end{array} } \right. $$

where $I$ is the entropy value; $N$ is the states of the system; $P_{{\text{n}}}$ is the probability of the n event in the corresponding state.

**Figure 11:**
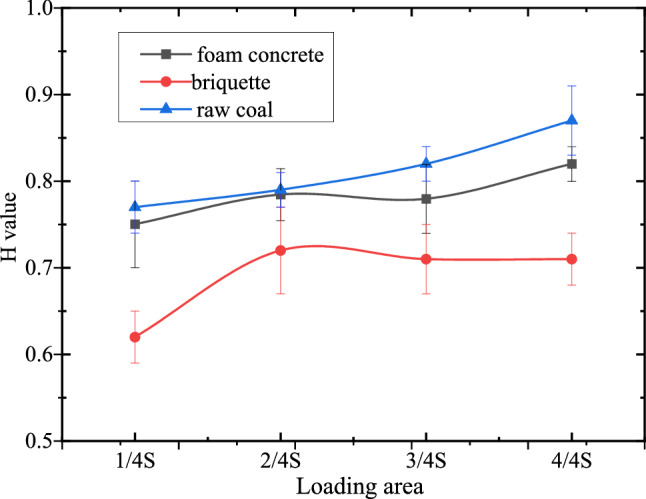
The relationship between the loading conditions and the *H* value.

According to the above formula, the entropy values of groups of raw coal, briquette and foam concrete specimens were normalized to obtain $H$ values under different loading conditions. The relationship between the loading conditions and the $H$ value was as shown in Fig. 11.

$H$ value of specimens was influenced by the combined of loading conditions and macroscopic central hole, which led to the emergence of the stress concentration zone and the change in temperature. It was found that $H$ values of three kinds of specimens increased with increasing of loading areas, but difference in grow trend. The $H$ value of raw coal increased linearly, while that of foam concrete and briquette gradually increased first and after that it had an approximate constant with the increase of loading area. The above result led to the conclusions that the material properties is at least one of the most important factors that lead to the change of temperature field of samples. Thermomechanical coupling effects of materials include thermoelastic effect, endothermic effect caused by gas escaping, and friction heat effect due to generation of a mass of cracks, the higher strength of specimens, the higher the temperature increase^48,49^. In addition, the average $H$ value of three kinds of specimens under local loading was generally smaller than that under loading at $S$, which reflected that the stress concentration under local loading was significant, resulting in more obvious non-uniform distribution of surface temperature. The experimental results indicated that temperature field can used to characterize the stress concentration and catastrophe of the specimen.

## Numerical simulation

### Development and validation of constitutive model

**Figure 12:**
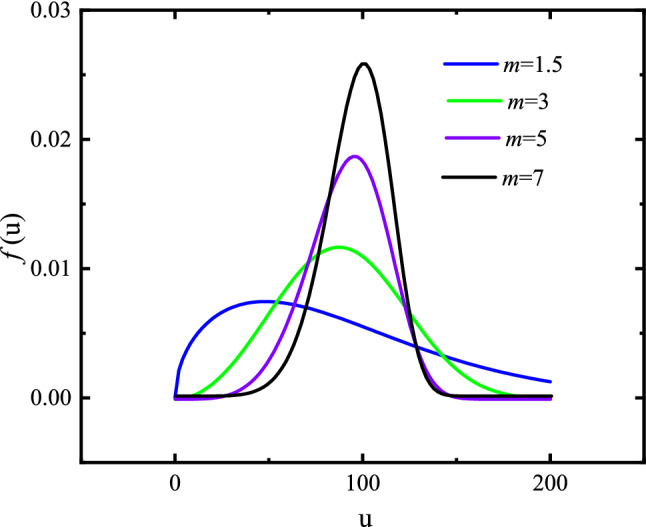
The Weibull probability density function for different m values.

In order to capture the heterogeneity of rock, its mechanical parameters, including the Young’s modulus and strength, are assumed to conform to the Weibull distribution^50,51^, which can be expressed as:

5 $$ f(u) = \frac{m}{{u_{0} }}\left( {\frac{u}{{u_{0} }}} \right)^{m - 1} \exp \left[ { - \left( {\frac{u}{{u_{0} }}} \right)^{m} } \right] $$

where *u* is the parameter of an individual element and *u*_0_ is related to the average element parameter, *m* is homogeneity index due to it reflected the distribution of element parameter. A value of the shape parameter, *m*, can be obtained from the sample as shown in Fig. 12.

**Figure 13:**
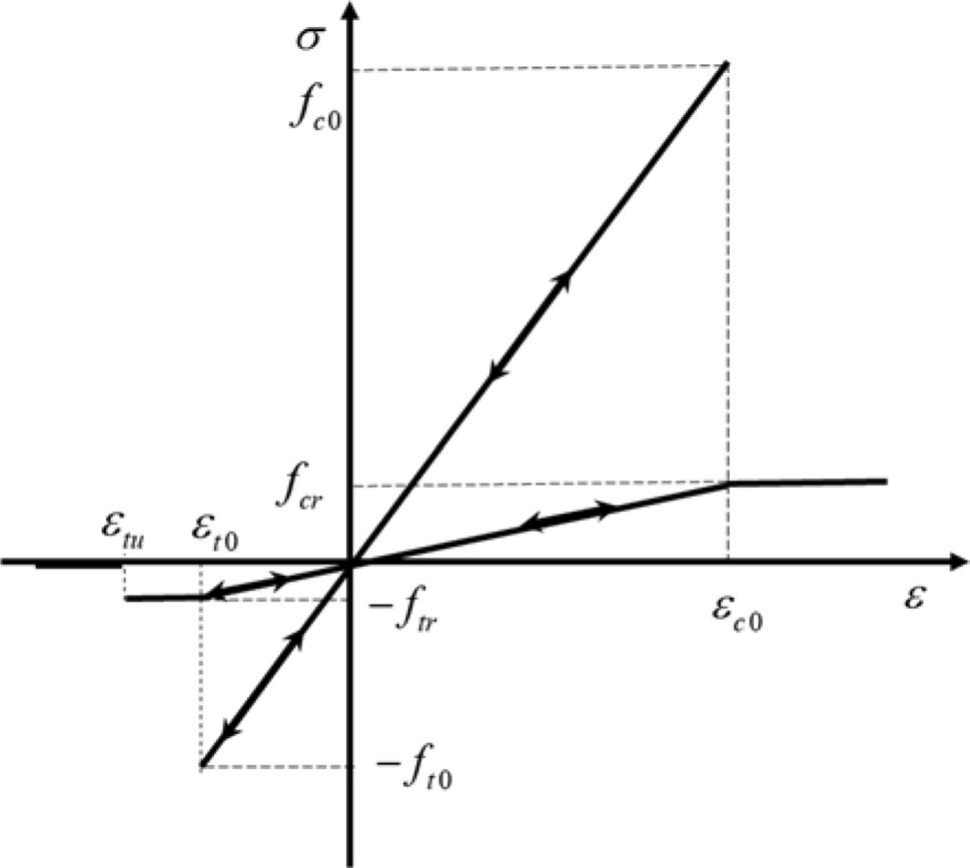
The constitutive law^51^.

The meso mechanics method was adopted to assume that every point in the rock mass satisfies the constitutive relation of linear elasticity. If the rock mass is damaged, its constitutive relation does not change, then the elastic modulus and strength begins to decrease. In this paper, maximum tensile-stress criterion and Mohr–Coulomb criterion are adopted as failure criteria^51^, as shown in Fig. 13, which can be expressed as:

6 $$ F_{1} \equiv - \sigma_{3} - f_{t0} \;\;{\text{or}}\;\;F_{2} \equiv \sigma_{1} - \sigma_{3} \frac{1 + \sin \varphi }{{1 - \sin \varphi }} - f_{c0} $$

7 $$ E = \left( {1 - D} \right)E_{0} $$

8 $$ D = \left\{ {\begin{array}{*{20}l} 0 \hfill & {F_{1} < 0,} \hfill & {\quad F_{2} < 0} \hfill \\ {1 - \left| {\frac{{\varepsilon_{t0} }}{{\varepsilon_{3} }}} \right|^{n} } \hfill & {F_{1} = 0,} \hfill & {\quad dF_{1} > 0} \hfill \\ {1 - \left| {\frac{{\varepsilon_{c0} }}{{\varepsilon_{1} }}} \right|^{n} } \hfill & {F_{2} = 0,} \hfill & {\quad dF_{2} > 0} \hfill \\ \end{array} } \right. $$

where $f_{t0}$ and $f_{c0}$ are uniaxial tensile strength and uniaxial compressive strengths of the unit, respectively. $E_{0}$ and $E$ are the elastic modulus before and after damage, respectively, $D$ represents the damage amount, $\varepsilon_{t0}$ and $\varepsilon_{c0}$ are tensile strain and maximum compressive strain corresponding to elastic limit respectively, $\varepsilon_{3}$ and $\varepsilon_{1}$ are tensile strain and compressive strain respectively, $n$ is a exponent of the power function, $F_{1}$ and $F_{2}$ are two functions reflecting stress states, it should be noted that under any stress condition, tensile damage is applied preferentially according to the elastic damage theory. C++ language can be used to write dynamic link base file to achieve a self-defined constitutive model.

The validity and accuracy of proposed models were tested by using the uniaxial compression test and by comparing the experimental results with the numerical results. The geometry of the sample was 50 mm (dia) × 100 mm (height), which was divided into 108,967 units, with 22,804 nodes. The size of Brazilian disc sample was 50 mm (dia) × 25 mm (height) and was divided into 46,104 units, with 8898 nodes. The parameters used in numerical simulation and experiment were shown in Tables 2 and 3.

**Table 2:** Main parameters in numerical simulation.

Homogeneity index	Mean elastic modulus (GPa)	Mean compressive strength (MPa)	Friction (°)	Compression–tension ratio	Poisson’s ratio	*f*_t0_ (MPa)	*f*_c0_ (MPa)	*ɛ*_t0_	*ɛ*_c0_
2	11.5	172.5	30.2	12.4	0.29	13.9	172.5	0.0012	0.015

**Table 3:** Main parameters of sandstone.

Density (g/cm^3^)	Elastic modulus (GPa)	Compressive strength (MPa)	Tension (MPa)	Cohesion (MPa)
2.17	8.4	34.5	2.4	6.2

**Figure 14:**
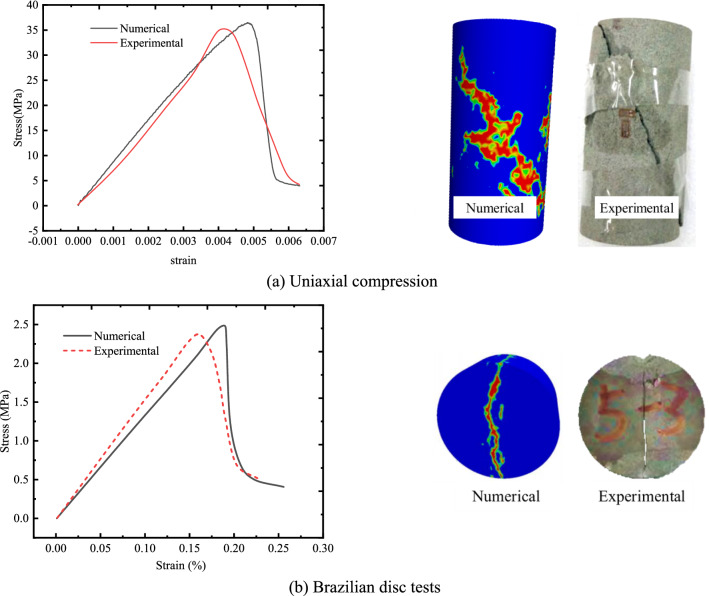
Comparison of numerical simulation results with experimental results.

As shown in Fig. 14, the comparison results between the numerical simulations and experimental study shows that proposed model can well simulate the progressive failure.

### Establishment of model

**Figure 15:**
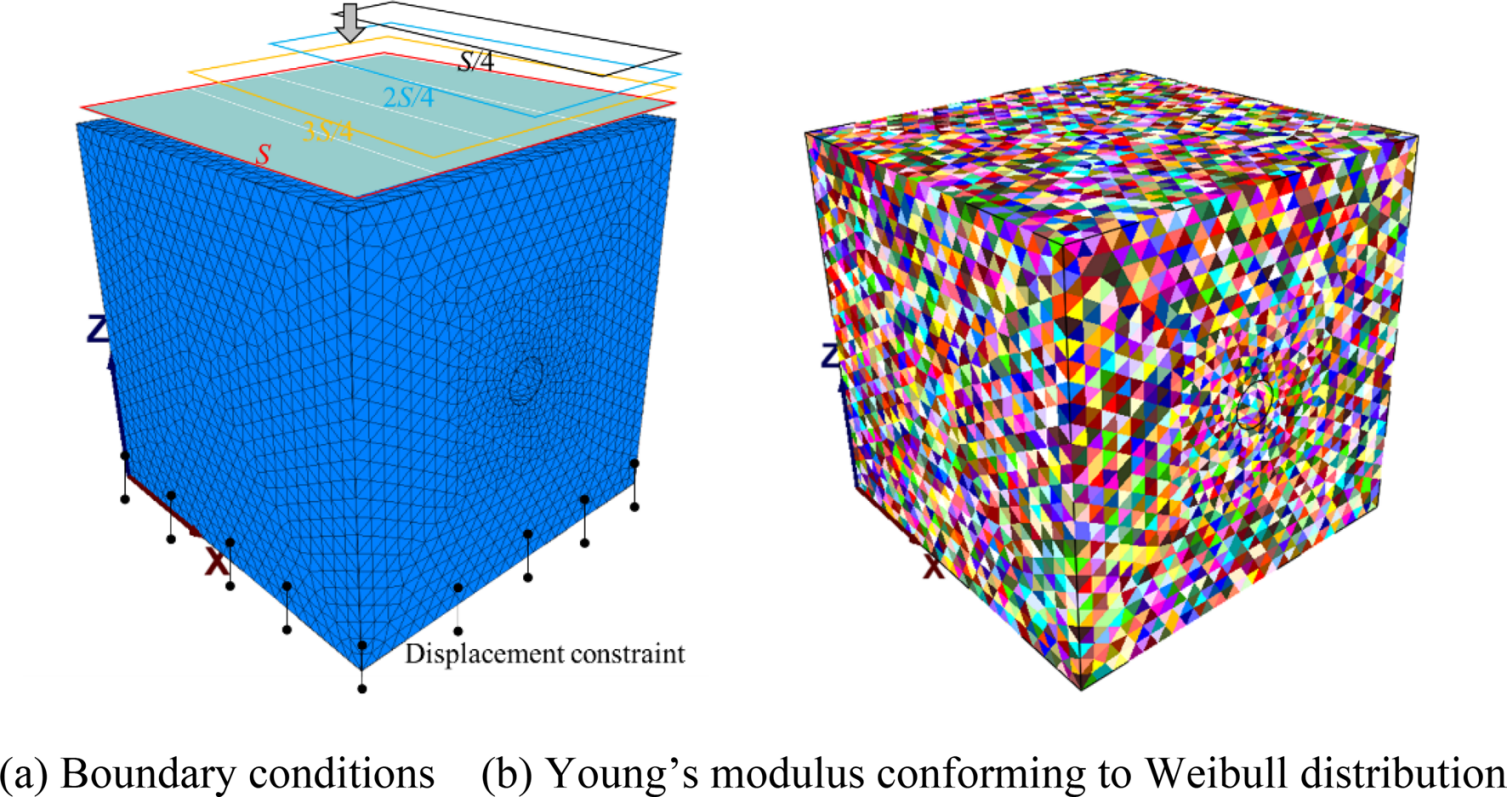
Boundary conditions and distribution of Young’s modulus.

To compare and confirm the experimental results, the groups of foam concrete were investigated in the numerical simulation. This paper adopts FLAC3D 5.0 (https://www.itascacg.com/) numerical simulation software, and the size of the numerical calculation model is the same as that of the experiments. As shown in Fig. 15, the model is divided into 109,025 units in total, with 20,492 nodes. The bottom of the model adopts displacement constraint, the varying loading areas are imposed at the top and the rest are free faces. The mean of the Elastic modulus and uniaxial compressive strength of elements in the specimens are specified to be 1.14 GPa and 15 MPa, respectively.

Table 4 showed parameters of the numerical conditions, the properties used in the model agreed with the experimental results conducted.

**Table 4:** Parameters of the specimens of foam concrete.

Density (g/cm^3^)	Mean Young’s modulus (MPa)	Internal frictional angle (°)	Poisson’s ratio	Cohesion (MPa)	*f*_t0_ (MPa)	*f*_c0_ (MPa)	ɛ_t0_	ɛ_c0_
0.723	1140	33.89	0.30	5.16	1.5	15	0.0013	0.013

### Crack initiation position

**Figure 16:**
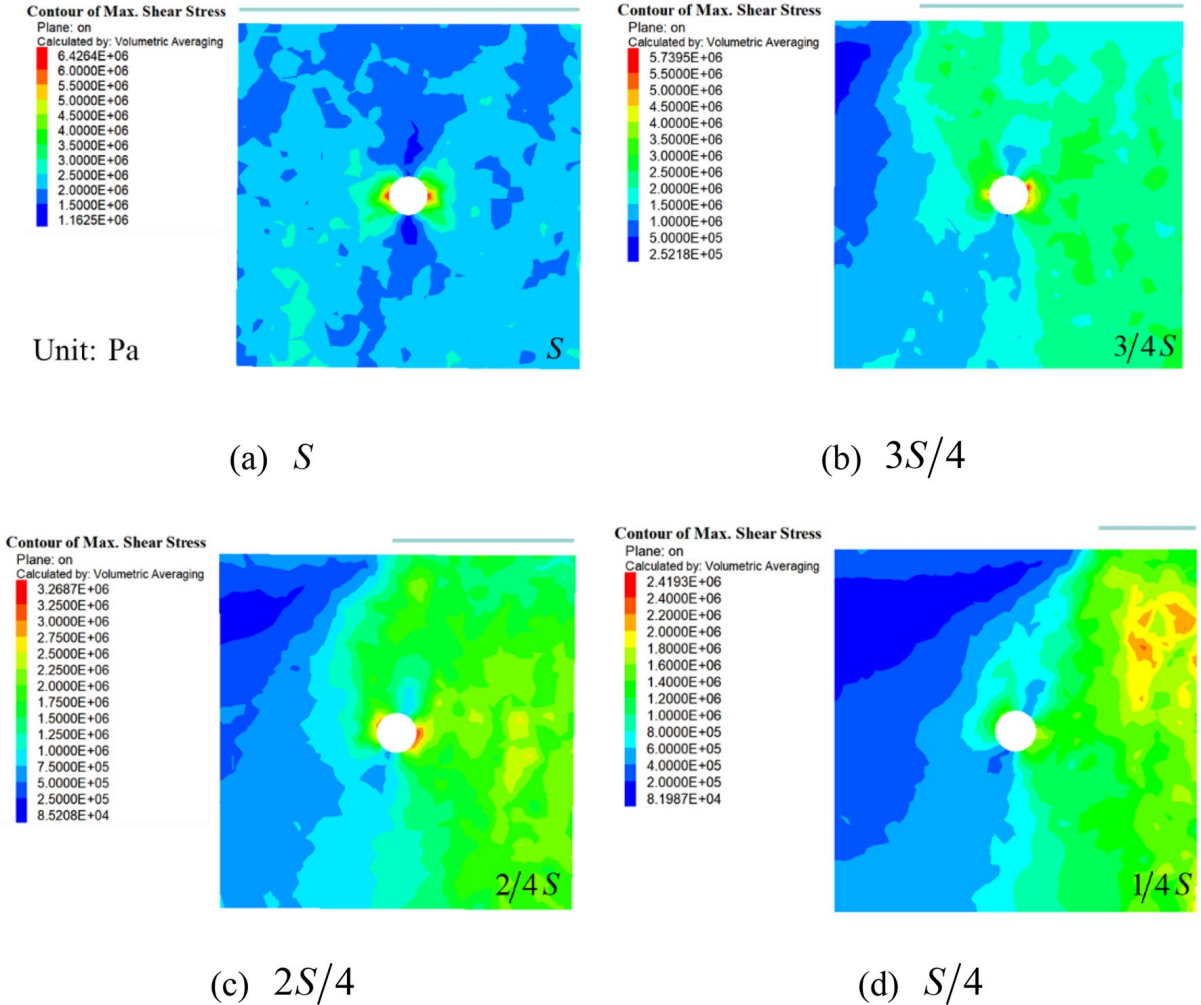
Shear stress under different loading areas.

Figure 16 showed the different stress distribution corresponding to different shear stresses with decreasing of loading areas, under full-area loading as shown in Fig. 16a, the cracks started around the hole and spread outward, which were distributed symmetrically around the center point of the hole. Then, under loading at ${{3S} \mathord{\left/ {\vphantom {{3S} 4}} \right. \kern-\nulldelimiterspace} 4}$ and ${{2S} \mathord{\left/ {\vphantom {{2S} 4}} \right. \kern-\nulldelimiterspace} 4}$, as shown in Fig. 16b,c, the cracks at the loading critical location grew downward and connected with the cracks around the hole, and then continued to extend towards the bottom of the specimen, in this case, the cracks were mainly distributed within the loading range. Last, under loading at ${S \mathord{\left/ {\vphantom {S 4}} \right. \kern-\nulldelimiterspace} 4}$, as shown in Fig. 16d, a nearly vertical crack was formed at the loading critical location, but no obvious failure occurred at the central hole.

**Figure 17:**
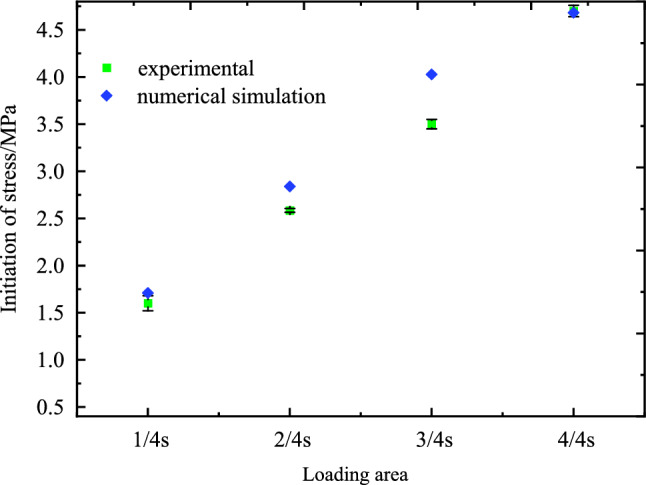
Start-up stress of under different loading areas.

The initiation stress was closely related to the elastic modulus of specimens during the damage process^52^, the initiation of internal cracks cannot be obtained directly due to limited conditions, thus the stress upon the initiation of cracks on the specimen surface was defined as the start-up stress. The relationship between the loading condition and the initiation stress was shown in Fig. 17, the initiation values of foam concrete specimens increased with increasing of loading areas. It was seen that, with the increase of the loading area, numerical simulation results were consistent with the experimental results, and the two were completely identical under loading area at $S$. Those results validated that the start-up stress defined was acceptable to characterize the crack state.

### Stress around the hole

**Figure 18:**
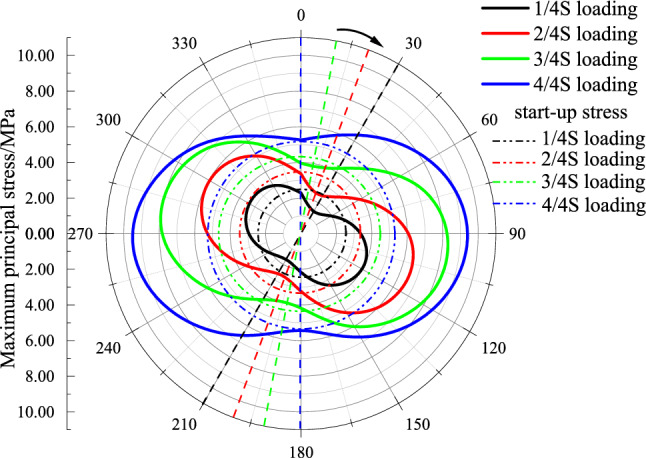
Distribution of stress around the hole.

The stress distribution of the model was discussed in detail at selected monitoring points, as shown in Fig. 18. Under the loading area at $S$, stress concentration occurred near the hole due to superposition of the central hole and the loading stress, the value of the stress gradually increased from the vertical to the horizontal direction, reaching 9.3 MPa at directions of approximately π/2 and 3π/2. Reduced it from $S$ to ${{3S} \mathord{\left/ {\vphantom {{3S} 4}} \right. \kern-\nulldelimiterspace} 4}$, the maximum stress around the hole decreased to 8.2 MPa, direction of which also deflected by 10°, and in this case, the cracks at the critical location occurred before that around the hole, which was because the crack initiation value was firstly reached, then cracks were generated around the hole and gradually connected with the cracks at the critical location. When the loading area was ${{2S} \mathord{\left/ {\vphantom {{2S} 4}} \right. \kern-\nulldelimiterspace} 4}$, the orientation of maximum stress around the hole change again, and the maximum stress went lower further. When the loading area was ${S \mathord{\left/ {\vphantom {S 4}} \right. \kern-\nulldelimiterspace} 4}$, the stress due to concentration was not high enough to cause cracks around the hole, a sudden failure occurred due to more tensile cracks forming along the discontinuities.

### Failure modes under local loading

Due to the difference of loading area, cumulative energy always resulted in failure, which was a complex process characterized by the initiation, propagation and perforation. Strain energy density can be utilized for calculating energy^53^, the equation was expressed as:

9 $$ U = \frac{1}{2E}\left[ {\sigma_{1}^{2} + \sigma_{2}^{2} + \sigma_{3}^{2} - 2v(\sigma_{1} \sigma_{2} + \sigma_{2} \sigma_{3} + \sigma_{1} \sigma_{3} )} \right] $$

where $E$ is elasticity modulus, $v$ is Passion's ratio, $\sigma_{1}$, $\sigma_{2}$ and $\sigma_{3}$ are major principal stress, intermediate principal stress and minor principal stress, respectively, $U$ is strain energy density.

**Figure 19:**
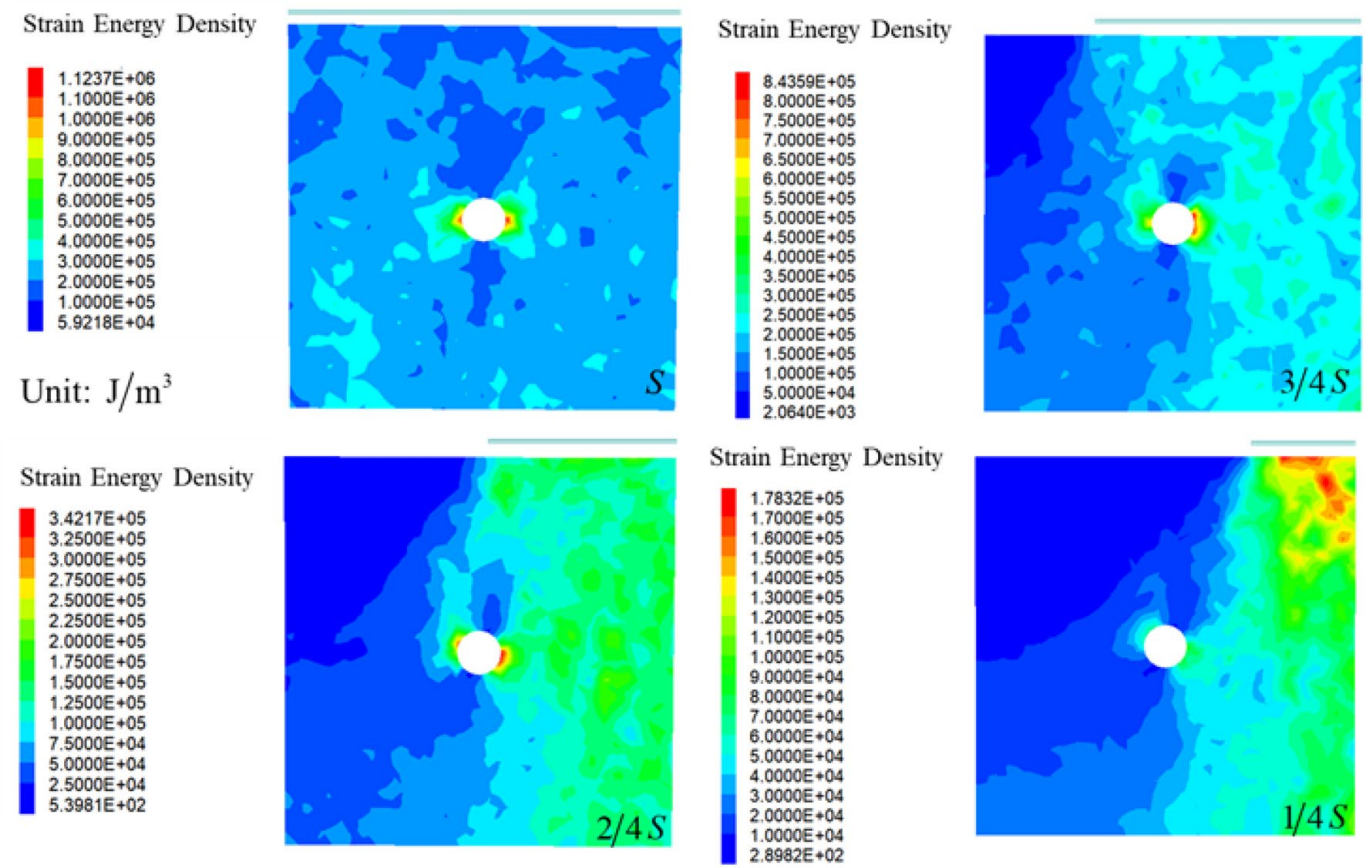
Distribution of strain energy density under different loading areas.

Failure modes were divided into three types based on strain energy density. The results were displayed as Fig. 19, rock failure occurred in the vicinity of the hole with the stress redistributed away from the hole when maintaining the loading area at $S$, the result indicated that the distribution of the SED was significant around the exist hole. After decreasing the loading area to ${{3S} \mathord{\left/ {\vphantom {{3S} 4}} \right. \kern-\nulldelimiterspace} 4}$ and ${{2S} \mathord{\left/ {\vphantom {{2S} 4}} \right. \kern-\nulldelimiterspace} 4}$, stress concentration occurred within loading area, cracks gradually contact to the exist hole and coalesce to form macroscopic cracks, indicating the distribution of the SED was significant in the loading area, but insignificant in the rest area. The maximum value of the SED was obtained on the loading area but not around the hole at ${S \mathord{\left/ {\vphantom {S 4}} \right. \kern-\nulldelimiterspace} 4}$. The results were agreed with experimental ones.

**Figure 20:**
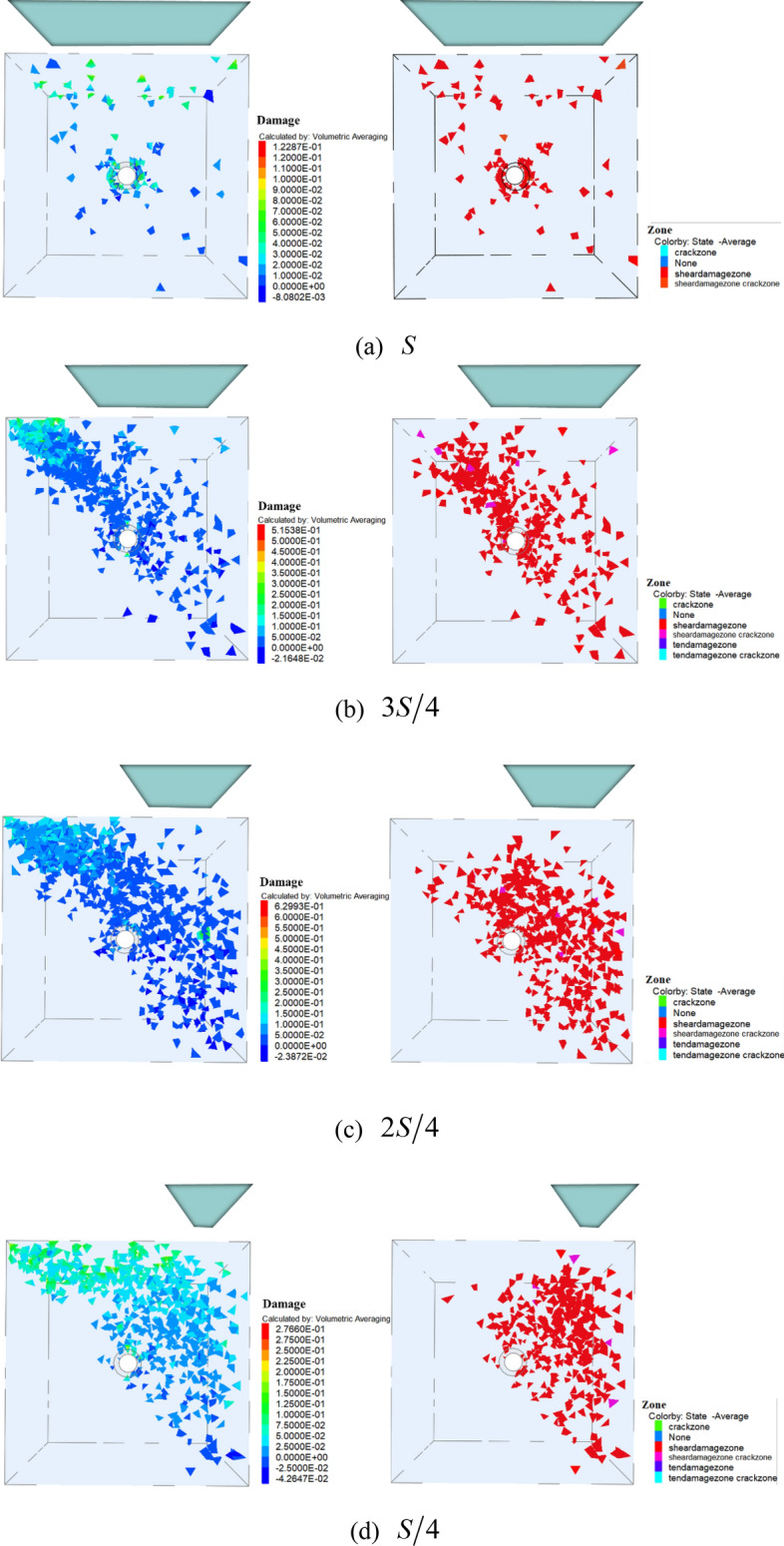
Damage of specimens under different loading areas.

Figure 20 showed the corresponding predicted damage zone and the corresponding predicted plastic zone during loading with varying loading areas. The plastic zone represented the failure zone of the specimen, while a self-defined constitutive model obtained from the secondary development was used to conduct quantitative express of the damage so as to show the whole process of progressive failure. The failure character of the specimen were shown respectively.

For the case of $S$, damage started to occur and cluster around the hole, where the plastic zone was basically the same as the damage zone, which was completely consistent with the conclusion obtained from the previous analysis. For the case of ${{3S} \mathord{\left/ {\vphantom {{3S} 4}} \right. \kern-\nulldelimiterspace} 4}$, ${{2S} \mathord{\left/ {\vphantom {{2S} 4}} \right. \kern-\nulldelimiterspace} 4}$, cracks appeared first at the critical location, afterwards, the micro-cracks propagated in this direction of the hole. Not until the cracks was growth and link up, do the microcracks start to propagate in the right diagonal direction. Finally, when many more cracks occurred which result in the failure of the whole specimen. It was worth noting that for the case of ${S \mathord{\left/ {\vphantom {S 4}} \right. \kern-\nulldelimiterspace} 4}$, the vertical cracks along the surface dominated the failure process of the specimen, indeed, it no longer passed through the central hole. It also was denoted that failure zone induced by tensile stress was shallower but the range was larger as the loading area decreased, the loading area significantly affected failure mode of the specimen.

## Conclusions

Considering of the influence of different loading areas, the mechanical properties of the specimen containing the central hole were analyzed as well as new constitutive models developed, the main conclusions can be drawn as follows:

1. Failure types can be divided into three modes depending on loading area. Under full-area loading, cracks appear first around the hole. With the loading area reduced to ${{S = 3S} \mathord{\left/ {\vphantom {{S = 3S} 4}} \right. \kern-\nulldelimiterspace} 4},{{2S} \mathord{\left/ {\vphantom {{2S} 4}} \right. \kern-\nulldelimiterspace} 4}$, cracks start to form at the loading critical location, gradually connect with ones at the central hole, under small loading area at $S = {S \mathord{\left/ {\vphantom {S 4}} \right. \kern-\nulldelimiterspace} 4}$, the cracks at the critical loading location grow almost vertically and would not pass through the macroscopic central hole.
2. Start-up stress is observed in the deformation field under different loading areas by using DIC, start-up stress and localized deformation energy decreased as loading areas decreased. The failure progress is important in regards to the prevention of mine disasters.
3. Temperature field is developed to analyze the progressive behaviors, increase in temperature of higher strength specimens was more obvious, meanwhile the stress concentration become more predominant under smaller loading areas, it is proved to be utilized to characterize of stress field and to predict the potential failure in advance.
4. The results of numerical simulation show that, a large amount of strain energy density accumulated will cause failure of the sample and induce initial strain energy release. The self-defined constitutive model established is implemented to characterize the damage, which is basically accord with that of the experiments.

As described above, the laboratory test and numerical simulation can be capable of well characterizing the failure progress of the specimen containing the central hole. Due to the limitation of the experimental machine conducted, the evolution of internal crack was not involved. In our future work, other numerical simulation method (the discrete element method) will be conducted to investigate the combined influence of loading conditions and macroscopic central hole on the failure mechanism under different load area based on the basic result presented in this paper.

The research presented in this study can be useful for predicting coal, rock and backfill failure and stress and energy evolution subjected to local loading, which are important for mining and civil engineering. The next stage is to take into account the effect of other factors such as the size, position, number of center holes, anisotropy, boundary effects and scale effects of specimens on the evolving damage behavior in different local loading conditions and compare it with experimental results. In future, other scenarios may be considered, for example, if the contact area is kept constant while loading conditions are changed, and compared with experimental results. Accurate prediction of the failure is of great significance in real rock mass or underground structures. The research results provide a theoretical basis for simulating the mechanical response and establishing an infrared early-warning technology. There is currently no ideal method for predicting the destruction, which should be further researched in the future.

## Data Availability

The data presented in this study are available on request from the corresponding author.
